# Spatially distinct microglia coordinate the response to photoreceptor injury in zebrafish

**DOI:** 10.64898/2026.09.04.749450

**Published:** 2026-09-10

**Authors:** Mikiko Nagashima, Lu Jiang, Sriram Garapati, Thanh Hoang, Peter F. Hitchcock

## Abstract

Microglia exhibit substantial molecular and functional heterogeneity, however, how the local tissue environment influences their responses to neuronal injury remains poorly understood. The zebrafish retina provides a unique opportunity to examine this relationship, because in addition to microglia within the retinal parenchyma, it contains a distinct population that resides within the subretinal space. In this study, we characterize the anatomical distribution and molecular heterogeneity of retinal microglia in zebrafish and examine their response to selective photoreceptor injury. Retinal microglia occupied distinct anatomical niches and exhibited diverse transcriptional states, including complement associated, proliferative, and apoc1/apoeb-enriched populations, suggesting that microglial heterogeneity reflects both tissue context and cellular. Following damage both subretinal and parenchymal microglia rapidly alter their morphology and migrate toward the injured site, resulting in their accumulation in the subretinal space. Microglia subsequently proliferate predominantly within this compartment. By 14 days post lesion, when photoreceptor regeneration is nearly completed, microglial morphology, distribution, and the number largely return to their unlesioned state. In contrast to the dynamic response of retinal microglia, response of microglia within the optic tectum was negligible. To investigate the mechanisms underlying the maintenance of these distinct microglial populations and their injury-induced responses, we examined retinal microglia in *csf1ra* mutants. In mutants lacking colony stimulating factor 1 receptor a (Csf1ra), parenchymal microglia are markedly reduced, whereas the number of subretinal microglia is preserved. Photoreceptor death following photolytic injury was comparable between *csf1ra* mutants and wildtype animals, and both parenchymal and subretinal microglia responded to the injury. However, proliferation of microglia in csf1ra mutants was severely impaired, and the normal redistribution of parenchymal microglia was not restored. These findings identify distinct anatomical and molecular features of retinal microglia and demonstrate that Csf1ra signaling differentially regulates microglial maintenance and injury-induced dynamics during photoreceptor regeneration.

## Full Text Availability

The license terms selected by the author(s) for this preprint version do not permit archiving in PMC. The full text is available from the preprint server.

